# Acute interstitial nephritis with only three doses of pantoprazole

**DOI:** 10.1002/ccr3.3059

**Published:** 2020-06-24

**Authors:** Subhankar Samal, Namrata Singhania, Saurabh Bansal, Girish Singhania

**Affiliations:** ^1^ Department of Hospital Medicine Ascension Columbia St. Mary's Hospital Milwaukee Wisconsin USA; ^2^ Department of Hospital Medicine Mount Carmel East Hospital Columbus Ohio USA; ^3^ Department of Internal Medicine University of Illinois at Peoria Peoria Illinois USA; ^4^ Department of Hospital Medicine Catholic Health Initiatives St Vincent Infirmary Little Rock Arkansas USA

**Keywords:** acute interstitial nephritis, acute kidney injury, proton pump inhibitors

## Abstract

Several antibiotics, proton pump inhibitors, and nonsteroidal anti‐inflammatory drugs can cause acute interstitial nephritis. This is not dose‐dependent, and recurrence can occur with a second exposure of the same drug. Stopping the culprit is critical for successful management.

## Graphical abstract

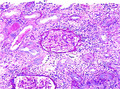

Acute interstitial nephritis is a common complication seen with use of proton pump inhibitors. This is the case of a young male who had severe acute interstitial nephritis with only three doses of pantoprazole.

A 21‐year‐old male presented to the emergency department for an elevated serum creatinine (Scr) level. He denied the use of any medications other than pantoprazole for the previous 3 days. The clinical examination was unremarkable. The diagnostic work‐up revealed blood urea nitrogen at 82 mg/dL (normal range [NR] 7‐20 mg/dL), Scr 15 mg/dL (baseline 0.6 mg/dL; NR 0.7‐1.2 mg/dL), normal creatine kinase level and no eosinophilia. Urinalysis showed non‐nephrotic proteinuria. Toxicology, HIV, hepatitis serologies, complement factors C3 and C4, and protein electrophoresis were unremarkable. Autoimmune workup including ANA, antistreptococcal, anti‐GBM, and ANCA antibodies was negative. Renal ultrasound was normal, and a renal biopsy was performed. There was a diffuse expansion of the interstitium with an accumulation of lymphocytes, plasma cells, and eosinophils with accompanying tubular inflammation consistent with acute interstitial nephritis (AIN) likely secondary to pantoprazole. (Figure 1) Immunofluorescence was negative for immune complex deposition. Pantoprazole was discontinued, and glucocorticoids (500 mg/d for 3 days, followed by 1 mg/kg/d) and hemodialysis were initiated. He recovered completely. Drugs like antibiotics, proton pump inhibitors, and nonsteroidal anti‐inflammatory drugs can cause AIN.^1^ This is not dose dependent, and recurrence can occur. Stopping the agent and early steroid use in selected cases confers better prognosis.^2^

**FIGURE 1:**
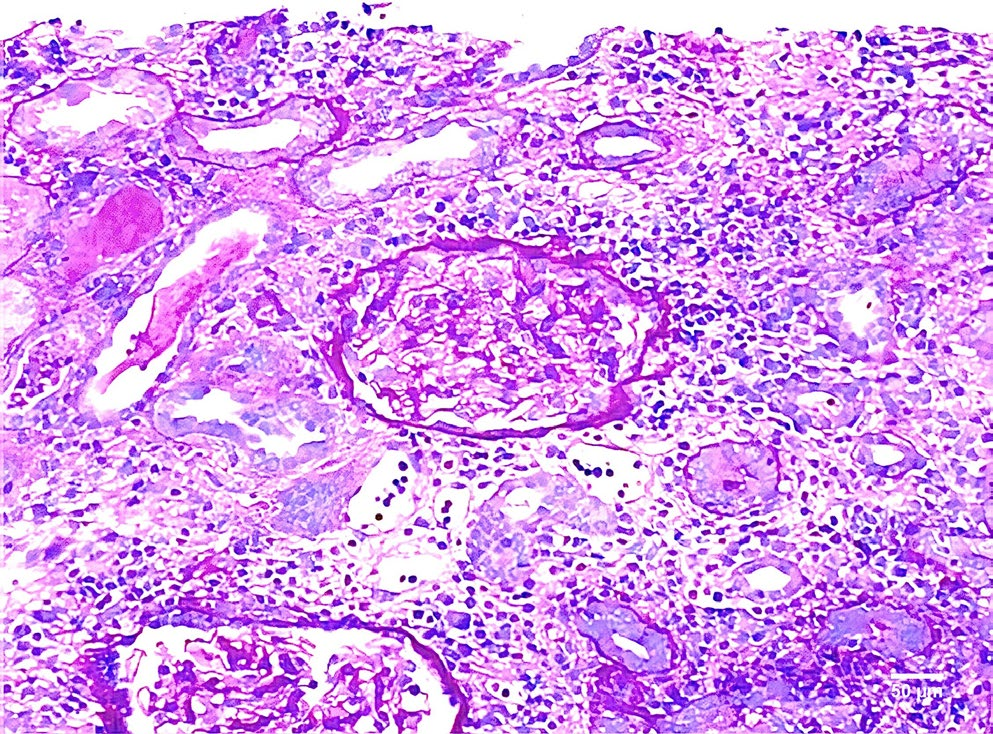
Renal biopsy showing diffuse expansion of the interstitium with lymphocytes, plasma cells, and eosinophils with accompanying tubular inflammation suggestive of interstitial nephritis

## CONFLICT OF INTEREST

None declared.

## AUTHOR CONTRIBUTIONS

SS and NS: have contributed equally to the manuscript. They wrote the manuscript and reviewed the literature. SB and GS: have reviewed the manuscript.
